# Flavonoid compound from *Agrimonia pilosa Ledeb* improves adipose insulin resistance by alleviating oxidative stress and inflammation

**DOI:** 10.1186/s12906-023-04114-5

**Published:** 2023-09-14

**Authors:** Tingwang Guo, Yun Pan, Lin Yang, Gang Chen, Jia Deng, Liancai Zhu

**Affiliations:** 1 Department of Gastroenterology, Children’s Hospital of Chongqing Medical University, National Clinical Research Center for Child Health and Disorders, Ministry of Education Key Laboratory of Child Development and Disorders, Chongqing Key Laboratory of Pediatrics, Chongqing, China; 2 College of Environment and Resources, Chongqing Technology and Business University, Chongqing, China; 3 Key Laboratory of Biorheological Science and Technology, Ministry of Education, College of Bioengineering, Chongqing University, Chongqing, China

**Keywords:** *Agrimonia pilosa Ledeb*, Flavonoid, Adipose, Insulin resistance, Oxidative stress, Inflammation, JNK

## Abstract

**Background:**

Researches and practice of traditional Chinese medicine indicated that *Agrimonia pilosa Ledeb* could improve insulin resistance (IR) and treat type 2 diabetes (T2DM). To reveal its underling mechanisms, we isolated Flavonoid component (FC) from *Agrimonia pilosa Ledeb* and elucidated its effects on glucose metabolism to improve IR by suppressing oxidative stress and inflammation.

**Methods:**

Adipocytes or mice IR model was established with overdosed glucose and insulin or high-fat diet. The uptake of 2-NBDG and glucose consumption were measured to verify insulin sensitivity in vitro and vivo. Reactive oxidative species (ROS) were detected by flow cytometry, and superoxide dismutase (SOD) activity as well as the malondialdehyde (MDA) content were also measured. Meanwhile, factors associated with insulin signal pathway including PPARγ, insulin receptor substrate-1 (IRS-1), GLUT4, and oxidative stress including NF-E2-related factor 2 (Nrf2), as well as the related inflammatory cytokines such as NF-κB, IL-1β, IL-6 and TNF-α were tested. Furthermore, the JNK/PI3K/Akt signal pathway was also explored.

**Results:**

FC extracted from *Agrimonia pilosa Ledeb* ameliorated the impaired glucose metabolism significantly. Further study indicated that FC could regulate the insulin signal pathway to improve insulin resistance. Moreover, it could upregulate PPARγ with the similar efficacy as pioglitazone (Piog) straightway. FC also decreased the endogenous ROS and MDA content, increased SOD activity and Nrf2 expression to facilitate oxidative homeostasis. It attenuated expression and secretion of inflammatory cytokines obviously. At last, our results indicated JNK/PI3K/Akt pathway was regulated by FC in adipocytes and adipose tissue.

**Conclusion:**

FC could ameliorate glucose metabolism and improve IR. It exerted these effects by suppressing oxidative stress and inflammation. FC from *Agrimonia pilosa Ledeb* has a good prospect to be drugs or functional foods for IR and T2DM.

**Supplementary Information:**

The online version contains supplementary material available at 10.1186/s12906-023-04114-5.

## Bcakground

Type 2 diabetes mellitus (T2DM) affected 536.6 million people in 20-79-year-olds in 2021 worldwide, while the number would reach to 738.2 million in 2045 [1]. Especially for middle-income countries, over 174 million people in China will suffer from diabetes by 2045 [1]. Considering the increasing disease burden, the treatment and prevention of this disease is urgently required. As a chronic metabolic disorder, it involves several pathogenic processes, mainly including insulin resistance (IR) and relative (rather than absolute) insulin deficiency. The impaired secretion and deficient action of insulin often coexist.

Oxidative stress is thought to be the main factor in the onset and progression of IR. Common Soil Hypothesis declares oxidative stress is the underlying pathogenic mechanism of IR and T2DM [2, 3]. Reactive oxidative species (ROS) derived from oxidative stress can decrease glucose consumption and disorder the insulin signaling pathway by disturbing GLUT to act on glucose transporting and accelerating serine phosphorylation of insulin receptor substrate-1 (IRS-1). ROS also triggers inflammatory cytokine secretion to worsen IR [4]. Consequently, anti-oxidative therapy can be an approach to preventing T2DM [4]. Furthermore, hyperglycemia and hyperinsulinemia are accompanied with systemic and tissue specific inflammation [5, 6]. For instance, elevated TNF-α and IL-6 have been reported in various IR states. Following c-Jun N-terminal kinase (JNK) and NF-κB activation mainly [5], inflammatory cytokines directly affect the insulin signal pathway such as disturbing IRS-1 and GLUT. By the way, they can also activate accumulation of free fatty acid reciprocally to worsen IR. In addition, JNK, as the common point of oxidative stress and inflammation, its phosphorylation can trigger the serine phosphorylation of IRS-1 and lead to IR. Series of reports clearly proved that ROS and inflammatory factors can phosphorylate JNK [7], and controlling JNK may play an indispensable role in preventing IR [8]. Taken together, alleviating oxidative stress and inflammation may be efficient and reliable in preventing IR and T2DM.

*Agrimonia pilosa Ledeb* is a tonic herb used to treat T2DM in traditional Chinese medicine. It includes flavonoids, flavone glycosides [9, 10], triterpenoids [11, 12] and agrimonolides. We found the flavonoid component (FC) was one of the most abundant components of *Agrimonia pilosa Ledeb* [13]. After tremendous endeavors, studies showed flavonoids had an anti-oxidative activity [14, 15], acted on immune system to exert an anti-inflammatory effect [16], and also had a positive effect on insulin sensitivity [17]. What’s more, it has been widely documented that *Agrimonia pilosa Ledeb* provided a considerable resistance to hyperglycemia and IR in insulin incubated-HepG2 cells [18] and high-fat diet-fed rats [19, 20]. Our previous study also indicated that extracts from *Agrimonia pilosa Ledeb* could reinforce insulin sensitization through PPARγ [12]. Therefore, in order to clarify whether FC from *Agrimonia pilosa Ledeb* has the therapeutic effect on IR, we collected FC and investigated its effects on 3T3-L1 adipocytes and C57BL/6 mice with IR induced by overdosed glucose and insulin. We detected the glucose consumption and uptake, determined the disturbed factors related to insulin signal, oxidative stress and inflammation, and explored JNK/PI3K/Akt signal pathway. This study will illuminate the mechanism of FC on improving IR and FC’s medicinal benefits.

## Methods

### Materials

*Agrimonia pilosa Ledeb* was obtained from Western Medicine City (Chongqing, China). The extraction process was conducted as previously described [13]. The total flavonoids concentration in FC was 316.53 ± 6.37 mg/g containing quercetin, quercitrin, vitexin, isovitexin, hyperoside, rutin, kaempferol, tiliroside, apigenin, catechinic acid, taxifolin and digitoflavone-7-O-glucoside mainly. The powder was stored at 4 °C.

Murine 3T3-L1 pre-adipocytes were purchased from ATCC. C57BL/6 mice were obtained from Children’s Hospital of Chongqing Medical University (China). The low-fat diet (LFD, 19% protein, 4% fat, 67% carbohydrate, and 10% calories from fat) and high-fat diet (HFD, 24% protein, 24% fat, 41% carbohydrate, and 45% calories from fat) were purchased from Shanghai SLAC Laboratory Animal CO. LTD. (Shanghai, China). DMEM, penicillin-streptomycin and FBS were purchased from HyClone. Bovine calf serum (BCS) was purchased from Gibco and BSA from Equitech-Bio. Paraformaldehyde, Oil Red O, 3-isobutylmethylxanthine (IBMX), dexamethasone (DEX) and insulin were purchased from Sigma-Aldrich (St Louis, MO). We got 2-[N-(7-nitrobenz-2-oxa-1, 3-diaxol-4-yl)amino]-2-deoxyglucose (2-NBDG) from Invitrogen. Glucose oxidase assay, ROS assay kit, total superoxide dismutase (SOD) assay kit with WST-1 and lipid peroxidation malondialdehyde (MDA) assay kit were purchased from Beyotime (Jiangsu, China). Trizol reagent was purchased from Invitrogen and reverse transcription system from Takara. SYBR Green was obtained from BIO-RAD. ELISA kits for insulin, IL-1β, IL-6 and TNF-α were purchased from 4 A Biotech (China). The antibodies of β-actin, p-IRS-1, GLUT-4, PPARγ, Nrf2, p-IκBα, NF-κB p65, t-/p-JNK 1/2, PI3K and p-Akt were purchased from Santa Cruz Biotechnology (Santa Cruz, CA, USA).

### Cell culture and adipocyte differentiation

Murine 3T3-L1 pre-adipocytes were induced to adipocytes based on the protocol as described [21]. Cells were maintained in DMEM (25.0 mmol/L glucose) supplemented with 10% BCS and 1% penicillin-streptomycin. At day 0, after reaching confluence, 3T3-L1 pre-adipocytes were treated with differentiation medium: DMEM (25.0 mmol/L glucose) supplemented with 0.5 mM IBMX, 1 µg/ml insulin, 0.25 µM DEX and 10% FBS for 48 h. For the following 48 h, cells were treated with DMEM (25.0 mmol/L glucose) supplemented with 1 µg/ml insulin and 10% FBS. Then treating with DMEM (25.0 mmol/L glucose and 10% FBS) for 6 days until more than 90% of cells were differentiated. After differentiation at 11th day, 3T3-L1 adipocytes could be used for Oil Red O staining and induced to establish IR model subsequently.

### Establishing 3T3-L1 adipocytes IR model

The model was established as described previously (Figure S1) [22]. After differentiation, the medium was replaced by DMEM (5.5 mmol/L glucose) with 1% BSA for 12 h. To establish IR model, the differentiated adipocytes were divided into LG group (DMEM containing 5.5 mmol/L glucose, 1% BSA and 10^− 9^ mol/L insulin), HG group (DMEM containing 25.0 mmol/L glucose, 1% BSA and 10^− 6^ mol/ L insulin), pioglitazone (Piog) group (10 µM Piog and DMEM containing 25.0 mmol/L glucose, 1% BSA and 10^− 6^ mol/ L insulin) and FC groups (25 µg/ml, 100 µg/ml or 400 µg/ml FC with DMEM containing 25.0 mmol/L glucose, 1% BSA and 10^− 6^ mol/L insulin). By glucose oxidase assay, the glucose concentration was analyzed according to the instruction. After 24 h, adipocytes were collected for further studies.

### Animals and drug treatments

All procedures were in accordance with the institutional guidelines for animal experimentation and approved by the Institutional Animal Care and Use Committee of Children’s Hospital of Chongqing Medical University (NO. 20,210,307,001). C57BL/6 mice (6 week) were housed under artificial lighting for 12 h/day and standard temperature (22–24 °C). Mice were fed with LFD or HFD randomly for 16 weeks. In the following 4 weeks, HFD mice were administered with Piog (5 mg/kg/day), FC (1.0, 2.5, 5.0, 7.5 or 10.0 mg/kg/day) or the same volume of PBS by intragastric gavage randomly each day. Body weight and food intake were recorded. After 4 weeks of drug treatment, the mice were anesthetized by inhalation of isoflurane and then euthanized by cervical dislocation. Serum insulin was detected by ELISA kit, blood samples and white adipose tissue (WAT) were collected and stored at -80 °C.

### Glucose uptake assay

In this study, 2-NBDG was used to monitor the glucose uptake as the method described previously [23]. Murine 3T3-L1 pre-adipocytes (8 × 10^3^/well) were plated in 96-well plate. Each group was divided into 3 wells: 100 µL KRB (Kreb Ringer Saline), 100 µL 50 µM 2-NBDG and 100 µL 50 µM 2-NBDG (containing 100 nM insulin). These wells were incubated at 37 °C for 30 min and the fluorescence intensity was detected with the excitation wavelength of 488 nm and emission wavelength of 520 nm. Results of 3 wells in each group were expressed as Fa, Fb, Fc. The calculation formula was (Fc-Fb)/(Fb-Fa).

### Intra-peritoneal glucose tolerance tests (iGTTs)

After 4 weeks of drug treatment, iGTTs of C57BL/6 mice were performed after overnight fasting. Mice were injected with 20% glucose solution at a dose of 2 g/kg, and then the blood glucose concentrations at 0, 15, 30, 60 and 120 min were measured by glucometer (OneTouch).

### Detecting intracellular ROS, activity of SOD, content of MDA and inflammatory cytokines

The fluorescence intensity of DCFH-DA (2’,7’-Dichlorodihydrofluorescein diacetate) in adipocytes was detected by flow cytometry. The activity of SOD was determined by WST-1 (Water-soluble tetrazolium salt-1) method at 450 nM. The intracellular content of MDA was determined at 532 nM. Both of them were detected by purchased kits.

The secretion of IL-1β, IL-6 and TNF-α was detected by ELISA according to the manufacturer’s protocol.

### RNA preparation and real-time PCR

Total RNA was isolated by Trizol reagent and 1 µg of isolated RNA was reverse-transcribed to cDNA by the reverse transcription system. According to the manufacturer’s protocol, Real-Time PCR with iQTM SYBR Green supermix was performed. Each reaction was performed in triplicate and GAPDH was used as the internal control gene. The fluorescence intensity was measured to monitor amplification of the genes. Then the comparative cycle time (CT) method determined the amount of target normalized to GAPDH and relative to a calibrator. The primers were shown in Table 1.

**Table 1:** Adipocyte specific primers for Real-Time PCR

Gene	Forward Primer	Reverse Primer
GAPDH	CAA GGT CAT CCA TGA CAA CTT TG	GGC CAT CCA CAG TCT TCT GG
IRS-1	ACC ATA ACC AGA GTG CCA AAG T	CGA GTA GGT GCT GAG AAG GTC T
GLUT4	CAT GGC TGT CGC TGG TTT C	AAA CCC ATG CCG ACA ATG A
JNK	ATG GAT TTG GAG GAA CGA ACT A	GAA GAC GAT GGA TGC TGA GAG
Nrf2	TCT TCC ATT TAC GGA GAC CCA	GAT TCA CGC ATA GGA GCA CTG
NF-κB	ATT CCG CTA TGT GTG TGA AGG	TGT GAC CAA CTG AAC GAT AAC C
IL-6	CTG CAA GAG ACT TCC ATC CAG TT	AAG TAG GGA AGG CCG TGG TT
TNF-α	CTG AGG TCA ATC TGC CCA AGT AC	CTT CAC AGA GCA ATG ACT CCA AAG

Abbreviations: IRS-1, insulin receptor substrate-1; JNK, c-Jun N-terminal kinase; Nrf2, NF-E2-related factor 2.

### Western blot

Proteins were extracted from cell or WAT lysis and their concentration was detected by BCA assay kit. Membranes were cropped along with the molecular weight marker to separate the target regions, and then incubated with antibodies. The bands were visualized by ECL and intensities were quantified using image analyzing software (Quantity One, Bio-rad).

### Statistical analysis

All data were presented as means ± SEM. A paired-samples T test was applied for two groups and one-way ANOVA followed by the Student-Newman-Keuls posttest for more than two groups by SPSS 19.0 software as appropriate. P value less than 0.05 in our study was considered to be statistically significant. All experiments were performed at least 3 times simultaneously.

## Results

### FC recovered the impaired insulin-stimulated glucose metabolism in 3T3-L1 adipocytes and C57BL/6 mice IR model

The morphology of pre-adipocytes was irregular, but adipocytes turned round after differentiation (Figure S2). Along with the inducing time extension, cells came into round and accumulated lipid droplets gradually. More adipocytes were observed in 10th day (Figure S2b) than those in 6th day (Figure S2a). Cells could be used to establish the IR model when the adipocytes reached to 80-90% (Figure S2b).

**Fig. 1:**
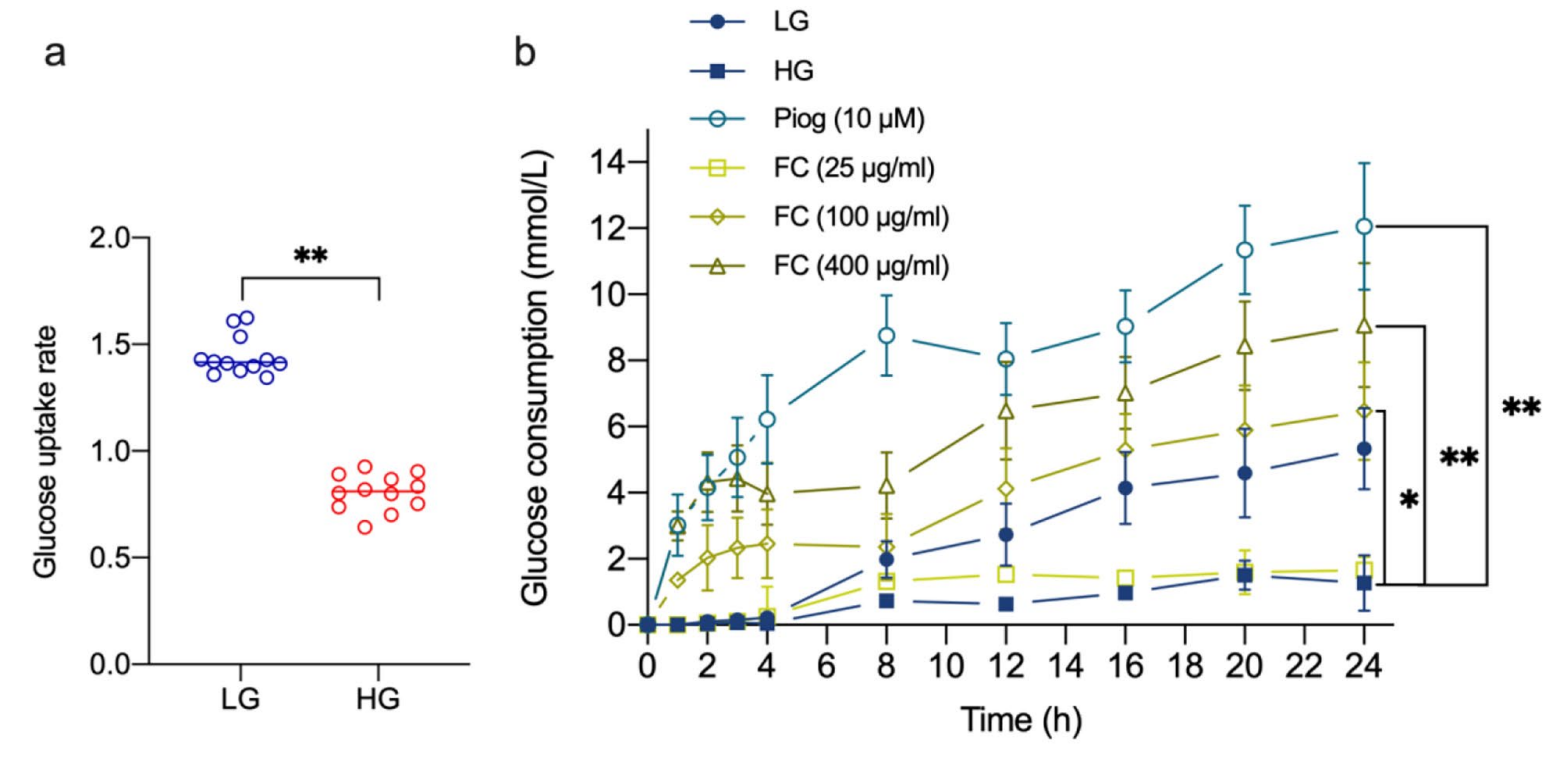
**a**, The 2-NBDG uptake rate in 3T3-L1 adipocytes. Insulin-stimulated glucose uptake was assayed with 2-NBDG. **b**, FC improved the glucose consumption of 3T3-L1 adipocytes with IR. Data are expressed as mean ± SEM (n = 12). * *p* < 0.05, ** *p* < 0.01 and *** *p* < 0.005. Piog, pioglitazone; FC, flavonoid component

As represented in Fig. 1a, the significant difference on the glucose uptake rate between the LG and HG group showed that the overdosed glucose and insulin impaired the capacity of glucose uptake. Furthermore, to illuminate the glucose consumption in 24 h, we detected the concentration of glucose in culture media (Fig. 1b). At 24 h, HG had a dramatically suppressed consumption in opposition to LG and Piog. However, the suppression was reversed by FC in a dose-dependent manner, especially at 100 µg/mL and 400 µg/mL, and was in accordance with the tendency of Piog.

**Fig. 2:**
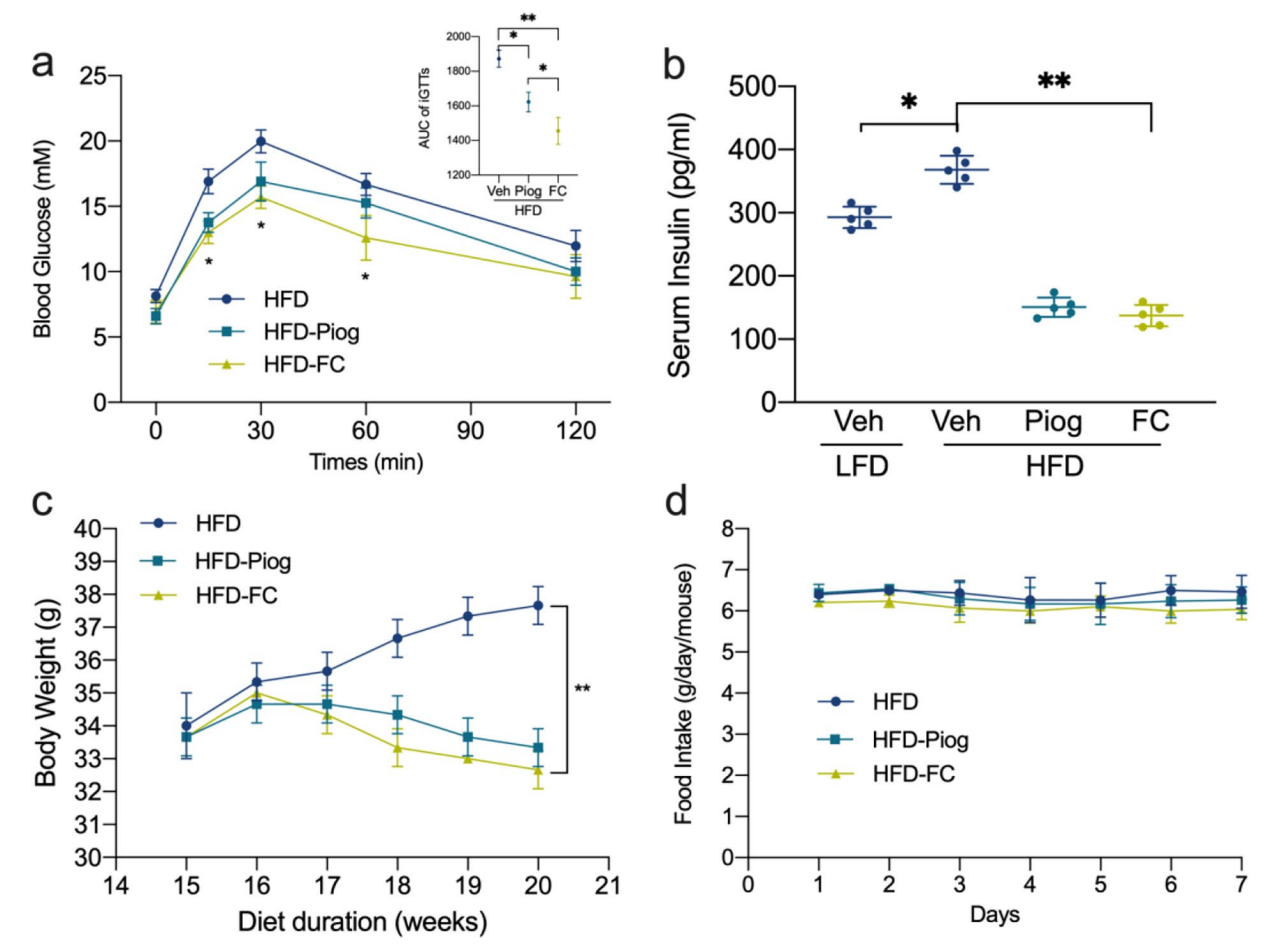
FC recovered the disturbed glucose metabolism and insulin resistance. iGTTs **(a)** were performed after starvation overnight after 20 weeks and AUC of iGTTs was calculated (a, the inserted figure). Serum insulin **(b)** after 20 weeks, body weight **(c)** from the 15th week to 20th week and food intake **(d)** in the last week were examined. Data are expressed as mean ± SEM (n = 6). * *p* < 0.05, ** *p* < 0.01 and *** *p* < 0.005. HFD, high fat diet; LFD, low fat diet; iGTTs, intra-peritoneal glucose tolerance tests; AUC, area under curve

We further evaluated the effect of FC on HFD-induced insulin resistance. Compared to LG and Piog, the plasma glucose concentration and area under curve (AUC) of intra-peritoneal glucose tolerance tests (iGTTs) were decreased statistically with FC at the dose of 5 mg/kg/day, especially at 15, 30 and 60 min after glucose loading (Fig. 2a and S3). FC with 7.5 and 10.0 mg/kg/day also showed significant decrease of plasma glucose concentration and AUC, while less obvious than that with 5 mg/kg/day. No significant difference was found between FC and HFD group under 1.0 and 2.5 mg/kg/day. Therefore, we chose FC of 5.0 mg/kg/day for the following experiments. In addition, HFD induced higher fed plasma insulin levels compared with LFD after 20 weeks. But FC and Piog decreased the induced hyperinsulinemia significantly, suggesting increased insulin sensitivity (Fig. 2b). Body weight changed significantly after administration (Fig. 2c), while no distinction on food intake was observed in the last week (Fig. 2d). These results clearly implied that HG and HFD had impaired glucose uptake and consumption under hyperglycemia and hyperinsulinemia, but FC could alleviate glucose metabolism disorders and improve IR obviously.

### FC improved the impaired insulin signal pathway

**Fig. 3:**
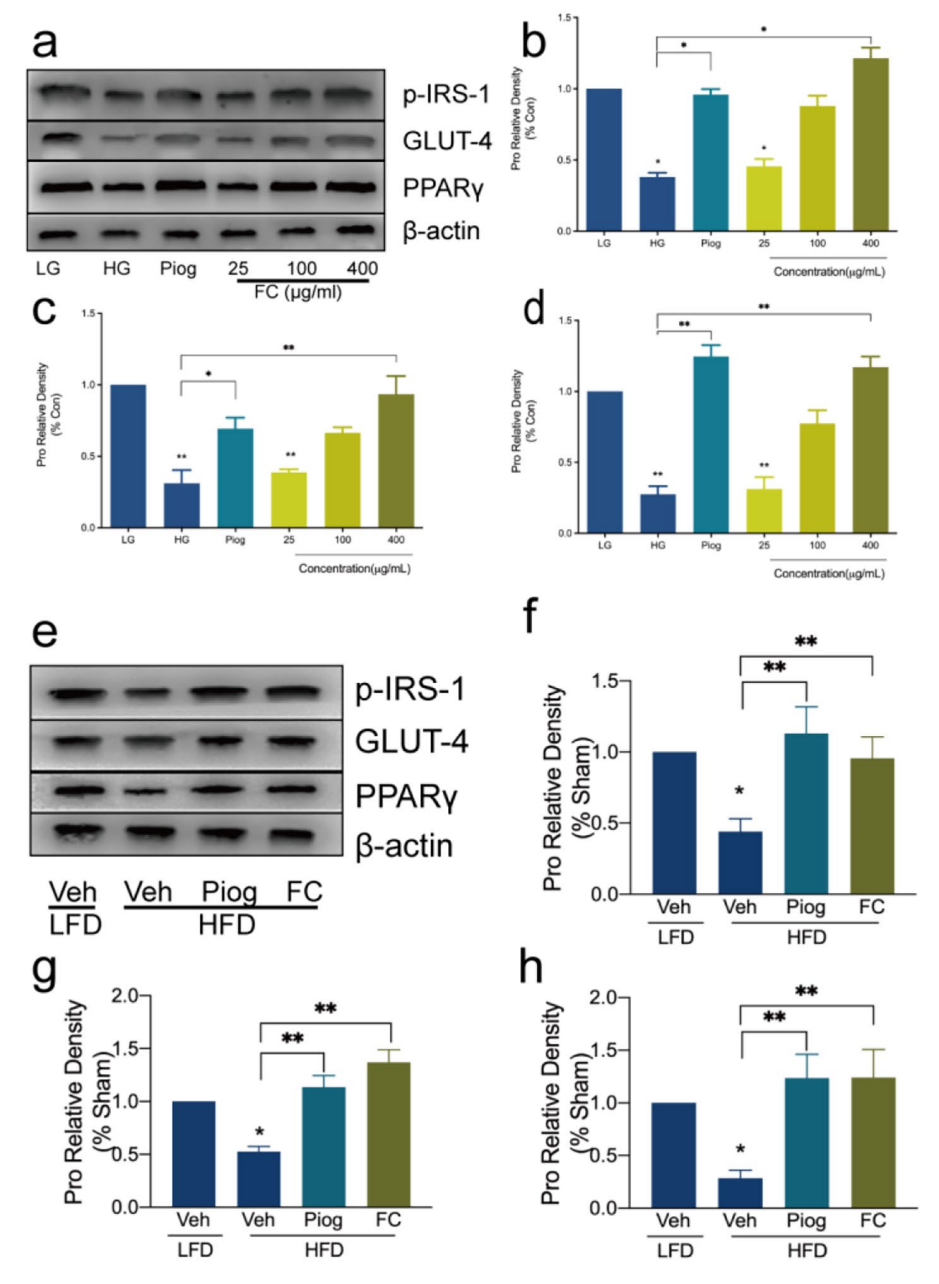
Effect of FC on the impaired insulin signal pathway in vitro and vivo. **a, b, c** and **d** referred to the protein expression of 3T3-L1 adipocytes. **e, f, g** and **h** referred to the protein expression of white adipose tissue (WAT) of C57BL/6 mice. a, b, e and f referred to the tyrosine phosphorylation of IRS-1 in 3T3-L1 adipocytes or C57BL/6 mice IR model. **a, c, e** and **g** referred to the expression of GLUT-4. **a, d, e** and **h** referred to the expression of PPARγ. The column graphs were the statistical results of band intensities and the intensities were quantified by Quantity One. Data are expressed as mean ± SEM (n = 6). * *p* < 0.05, ** *p* < 0.01 and *** *p* < 0.005

Insulin signal regulates glucose and insulin metabolism of adipocyte tissue, and is affected by the tyrosine phosphorylation of IRS-1 and translocation of GLUT-4 [24]. Herein, in 3T3-L1 adipocytes IR model, the tyrosine phosphorylation (Fig. 3a and b) and gene expression (Figure S4a) of IRS-1 were attenuated in HG group. Not surprisingly, the high level of glucose and insulin also induced the impaired protein (Fig. 3a and c) and gene (Figure S4b) expression of GLUT-4 which suppressed glucose translocate and uptake. Consistent with the observations in vitro, the tyrosine phosphorylation of IRS-1 (Fig. 3e and f and Figure S5a) and GLUT-4 (Fig. 3e g and Figure S5b) were dramatically decreased by HFD. However, FC improved the disordered signal pathway markedly.

Piog, a PPARγ agonist, improves insulin sensitivity and metabolic profile. So Piog could not only facilitate expression of PPARγ, but also give rise to the elevated p-IRS-1 and GLUT-4 in IR model (Fig. 3). In our study, we found FC from *Agrimonia pilosa Ledeb* also increased PPARγ (Fig. 3a, d and e h), p-IRS-1 and GLUT-4 expressions significantly as Piog. These results were in accord with the upward glucose consumption in Fig. 1 and blood glucose levels in Fig. 2. Results showed that IR model had impaired expression related to insulin signal pathway, but FC had an effect on promoting their expression and enhanced insulin sensitivity to improve IR and prevent T2DM.

### FC abolished the induced oxidative stress in IR model

**Fig. 4:**
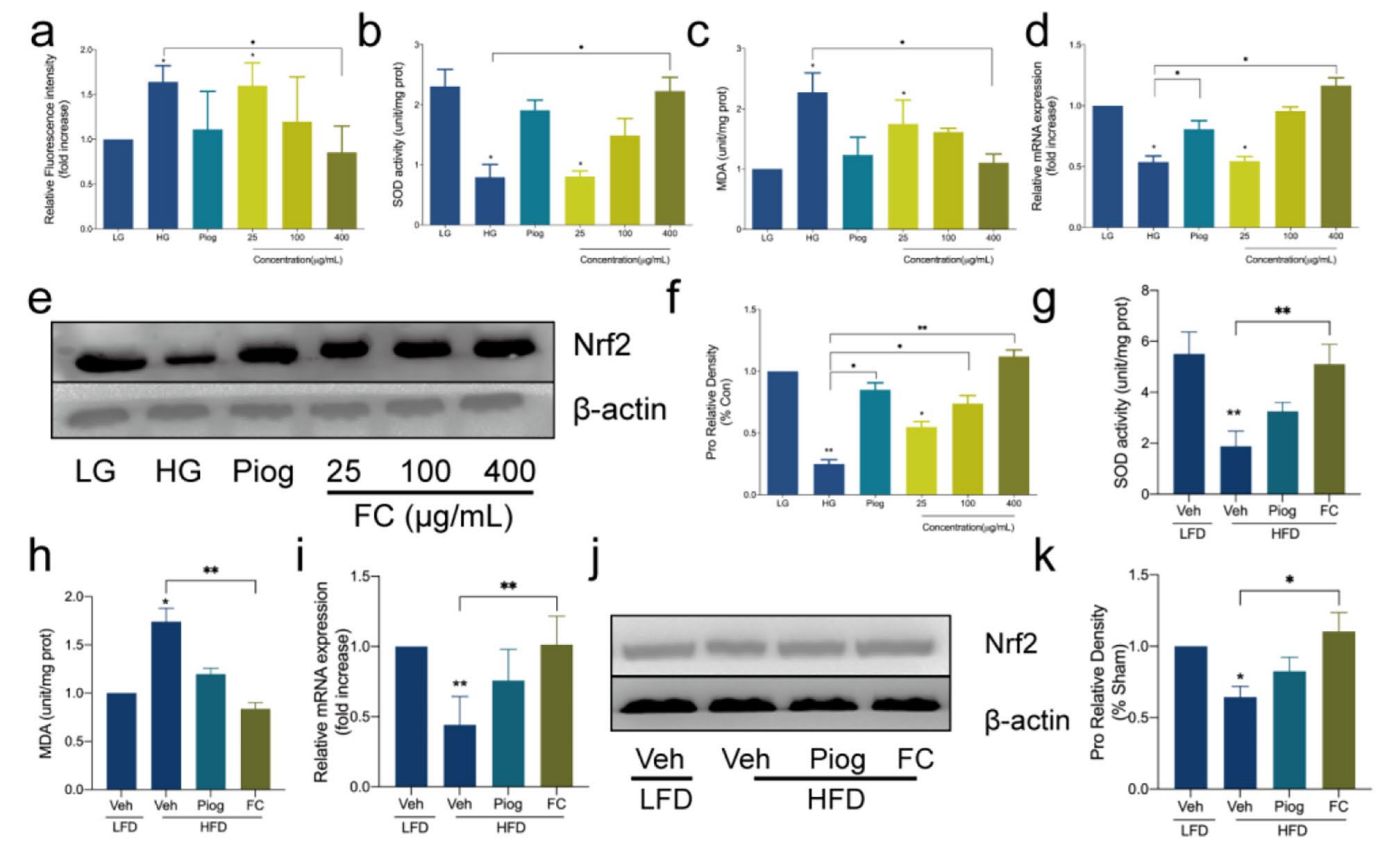
Effect of FC on the oxidative stress in vitro and vivo. **a** referred to intracellular ROS. **b** and **g** referred to the activity of SOD. **c** and **h** referred to the content of MDA. **d** and **i** referred to the gene expression of Nrf2. **e, f, j** and **k** referred to the protein expression of Nrf2 in 3T3-L1 adipocytes or the white adipose tissue (WAT) of C57BL/6 mice IR model. Data are expressed as mean ± SEM (n = 6). * *p* < 0.05, ** *p* < 0.01 and *** *p* < 0.005. SOD, superoxide dismutase; MDA, malondialdehyde

The occurring of IR promotes the augment of ROS which also aggravates IR further by disturbing insulin signal pathway. Under the excessive concentration of glucose and insulin, ROS of adipocytes in HG increased and showed a significant difference compared with LG (Fig. 4a). Additionally, HG and HFD had considerable effects on the SOD activity (Fig. 4b g) and MDA level (Fig. 4c h) in vitro and vivo. Conversely, FC abolished the induced oxidative stress compared with IR groups, although difference on effect of Piog on these factors did not achieve the significant level.

Nrf2, a transcription factor, involves in the critically important cellular defense mechanisms that maintains intracellular redox homeostasis and minimizes oxidative damage. Our research exhibited that its expression was remarkably depressed in HG group (Fig. 4d, e and f) and HFD group (Fig. 4i, j and k), respectively. At dose of 100 and 400 µg/mL in vitro, or 5 mg/kg/day in vivo, FC could increase Nrf2 expression significantly and were much higher than Piog. Based on these results, FC reduced intracellular level of oxidative stress, increased anti-oxidation, and improved IR and T2DM.

### FC ameliorated expression and secretion of factors related to inflammation

**Fig. 5:**
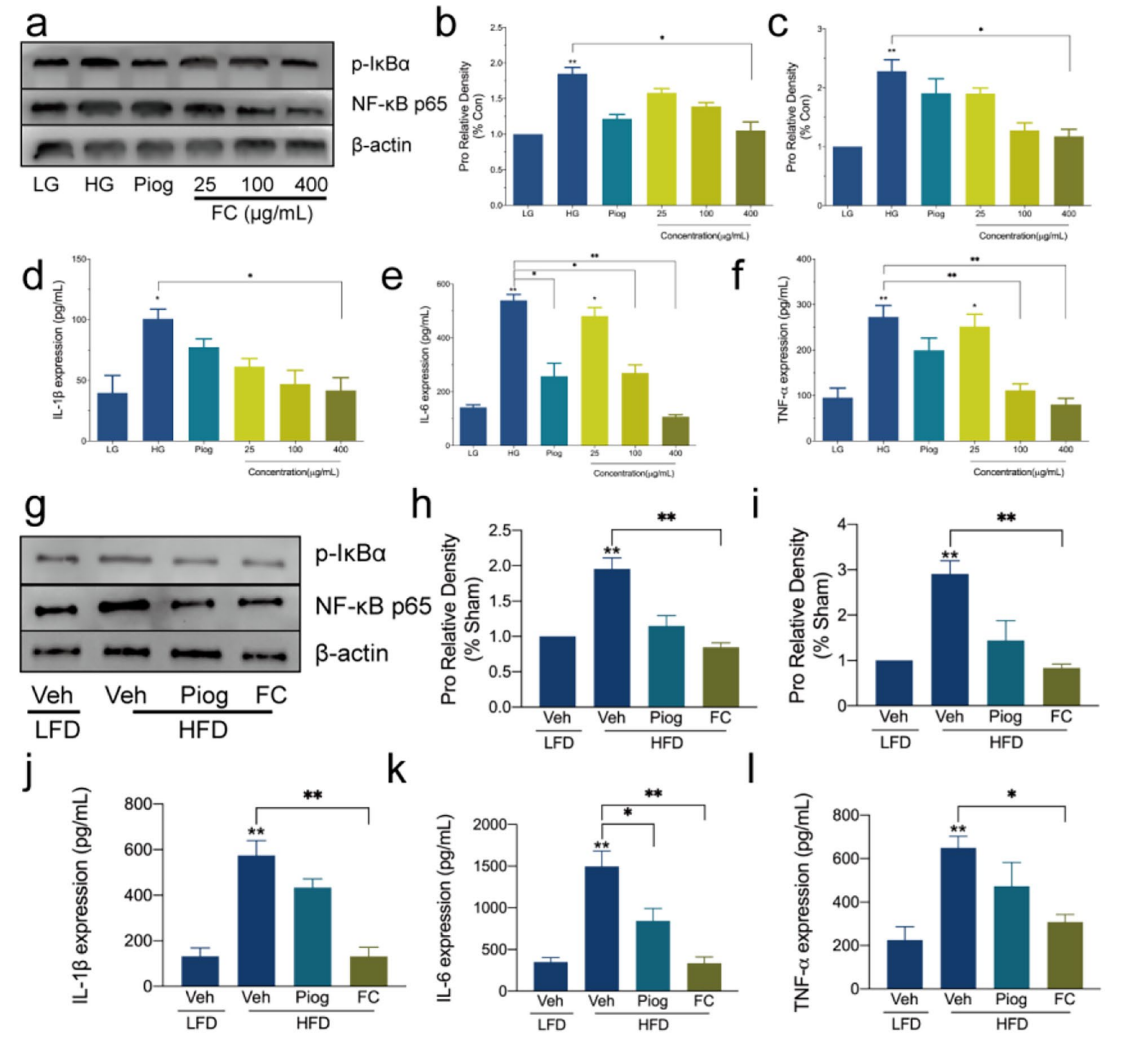
Effect of FC on the inflammatory stress in 3T3-L1 adipocytes and C57BL/6 mice IR model. **a** and **b** referred to the threonine phosphorylation of IκBα in 3T3-L1 adipocytes. **a** and **c** referred to the expression of NF-κB p65 in 3T3-L1 adipocytes. **g** and **h** referred to the threonine phosphorylation of IκBα in the white adipose tissue (WAT) of C57BL/6 mice. **g** and **i** referred to the expression of NF-κB p65 in the WAT of C57BL/6 mice. **d, e, f, j, k** and **l** referred to the expression and secretion of IL-1β, IL-6 and TNF-α in 3T3-L1 adipocytes and the serum of C57BL/6 mice. Data are expressed as mean ± SEM (n = 6). * *p* < 0.05, ** *p* < 0.01 and *** *p* < 0.005

With IR, the content of inflammatory factors was relatively high in 3T3-L1 adipocytes and C57BL/6 mice, but FC could suppress them (Fig. 5, S6 and S7). Among these inflammatory factors, NF-κB is particularly noteworthy, which plays a pivotal role in the inflammatory stress, and is activated dramatically in IR models. Therefore, the threonine phosphorylation of NF-κB and IκBα were detected firstly. Hyperglycemia and hyperinsulinemia gave rise to higher elevated threonine phosphorylation in opposition to LG and LFD group (Fig. 5a, b and c g, 5 h and 5i). What’s more, as a proinflammatory cytokine, NF-κB initiated the following response including the expression and secretion of IL-1β (Fig. 5d and j), IL-6 (Fig. 5e and k) and TNF-α (Fig. 5f L) significantly. However, compared with HG or HFD group, FC decreased the expression and secretion of these cytokines dramatically, Piog did not show significant difference. These observations strongly supported that FC could suppress inflammation and become the critical role in preventing IR.

### FC imped the induced-JNK/PI3K/Akt in adipocytes and adipose tissue

**Fig. 6:**
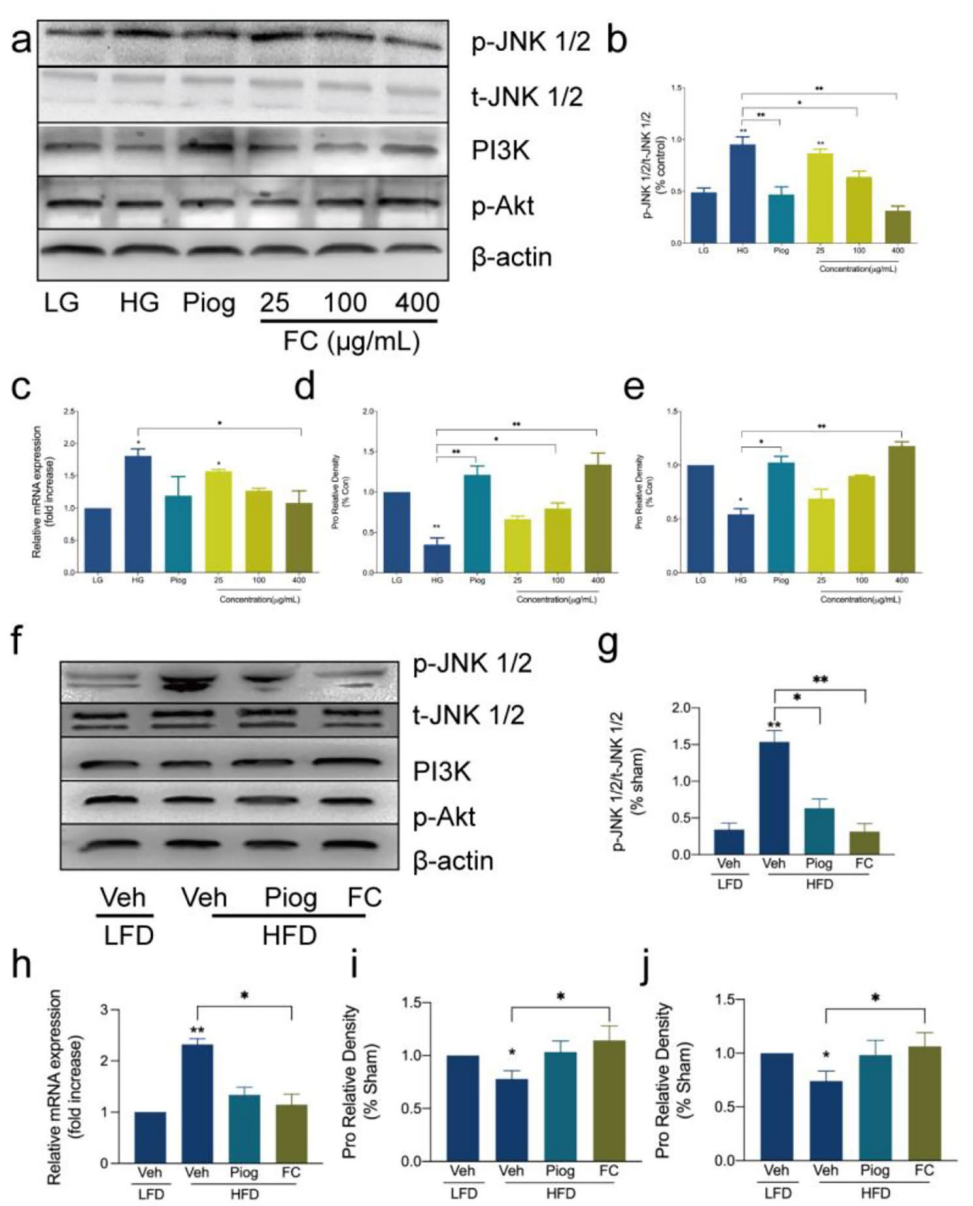
Impact of FC on JNK/PI3K/Akt pathway triggered by insulin resistance in adipocytes and the white adipose tissue (WAT) of mice. **a, b, c, d** and **e** referred to the expression in 3T3-L1 adipocytes. **f, g, h, i** and **j** referred to the expression in the WAT of C57BL/6 mice. **a, b, f** and **g** referred to the threonine phosphorylation of JNK. **c** and **h** referred to the gene expression of JNK. **a, d, f** and **i** referred to the expression of PI3K. **a, e, f **and **j** referred to the tyrosine phosphorylation of Akt. Data are expressed as mean ± SEM (n = 6). * *p* < 0.05, ** *p* < 0.01 and *** *p* < 0.005

The toxicity from hyperglycemia and hyperinsulinemia was inevitable responsible for the upward factors associated with inflammation and oxidative stress in the previous work and aforementioned experimental results. In this case, JNK/PI3K/Akt pathway in adipocytes and WAT was analyzed. First of all, JNK can be induced by oxidative stress and inflammation, and is extremely extraordinary in adipocytes or adipose tissue with IR [25]. Here, the threonine phosphorylation and expression of JNK were significantly different in opposition to un-treated groups in vitro and vivo (Fig. 6a, b, c and f g and 6 h). Furthermore, the disorder also had a great impact on PI3K and Akt as JNK. They were suppressed markedly compared with LG and LFD (Fig. 6a, d, e, f, i and j). Strikingly, FC from *Agrimonia pilosa Ledeb* prevented the over-expressed and phosphorylated JNK, facilitated the indispensable expression of PI3K and Akt, and was also stronger than Piog. In conclusion, the high level of glucose and insulin contributed to the activated JNK/PI3K/Akt, but FC could alleviate this signal pathway. This might be the feasible approach to suppress oxidative stress and inflammation in order to prevent IR.

## Discussion

The occurrence and development of T2DM involved factors and signal pathways including IR, oxidative stress, inflammation et al. Studies have reported that oxidative stress played a causal role in IR and T2DM, and inflammation also contributed to the disease occurrence [26]. Therefore, the anti-oxidative and anti-inflammatory drugs may be a promising approach in improving IR and preventing T2DM. In our previous study, we found *Agrimonia pilosa Ledeb* had the effect of anti-oxidation and anti-inflammation against hyperglycemia and hyperinsulinemia. Here, we extracted FC from *Agrimonia pilosa Ledeb* and studied its effects on improving IR in 3T3-L1 adipocytes and C57BL/6 mice with IR. Our results indicated FC could ameliorate the glucose metabolism and enhance insulin sensitivity via preventing oxidative stress and inflammation though JNK/PI3K/Akt signal pathway in vitro and vivo.

Adipose tissue is known to be the common site involved in IR, T2DM and cardiovascular. Either local and systemic adipose tissue can release ROS [4, 27] and multiple inflammatory cytokines [28]. Therefore, in this study, adipocytes and adipose tissue were exposed to overdosed concentration of glucose and insulin chronically to cause insult and used to establish IR model. As expected, glucose metabolism in IR groups was significantly impaired, and surprisingly restored by FC from *Agrimonia pilosa Ledeb*. Furthermore, deficiency in insulin pathway, mainly PPARγ/IRS/GLUT, which leads to the disruption of insulin and glucose homeostasis has considerable effects on the development of IR [29]. It should be noted that PPARγ has received a substantial attention owing to its effect on reducing hyperglycemia by increasing insulin sensitivity and decreasing hepatic glucose production [30]. The elevated PPARγ significantly ameliorates insulin sensitivity in mice with high fat diets and patients with T2DM by facilitating IRS and GLUT [29]. IRS-1, a principal substrate of receptor for insulin, plays an important role in insulin signal pathway. Inflammatory cytokines can induce its serine and inhibit tyrosine phosphorylation to reduce biological effect of insulin and to deteriorate IR, so the lower expression of IRS-1 predicts IR [31]. Another major factor contributing to the impaired glucose transport is the down-regulated GLUT4 [6]. It is the most abundant facilitative glucose transporter in adipose tissue [32]. These series of results clearly reveled that PPARγ, IRS-1 and GLUT4 were significantly inhibited in IR, but FC improved the inhibition in a dose-dependent manner. Taken these together, the disordered glucose and insulin contributed to the IR of adipocytes and adipose tissue, while FC facilitated glucose intolerance and reinforced insulin sensitivity.

Oxidative stress and inflammation play indispensable roles in the initiation and progression of IR. ROS can intervene the insulin signal pathway which causes resistance to insulin action by accelerating serine and inhibiting tyrosine phosphorylation of IRS-1. By the way, NF-κB can also be activated by ROS and then aggravates IR. Whereafter, the expression and secretion of inflammatory cytokines are accelerated. With the emergence of oxidative stress, antioxidant enzymes and oxidized products are altering. The content of MDA, the lipid oxidative end product, represents the level of oxidative insult. With respect to the endogenous anti-oxidative defense, SOD can decrease the oxidative stress and inflammation [26]. Particularly, Nrf2 can achieve self-protection through nuclear accumulating and up-regulating of ARE-linked genes expression, such as SOD and CAT. So the activation of Nrf2 is indispensable to keep cellular homeostasis and prevent the initiation of IR and T2DM [33]. The obtained results indicated that FC significantly depressed ROS and MDA in vitro and vivo. The activity of SOD and expression of Nrf2 were promoted to achieve an enhanced capacity of anti-oxidative stress. An increasing level of anti-oxidative stress and decreasing endogenous ROS might be essential to the effect of FC on preventing IR.

Another initiation factor, inflammation [5], can also induce IR by suppressing insulin signal pathways. NF-κB, as a nuclear transcription factor, regulates inflammatory cytokines, adhesion molecules and chemotactic cytokines to influence inflammatory reactions and immunological processes [34]. Activation of NF-κB is initiated by stimuli, such as FFAs and cytokine receptors, and also depends on the threonine phosphorylation of IκB [34]. The synthesis of IL-1β, TNF-α and IL-6 is mediated by NF-κB, and they activate NF-κB in turn. Meanwhile, the above cytokines also can be activated by ROS. Especially in adipose tissue, IL-1β and TNF-α aggravate IR though activating various signal transduction cascades which inhibit insulin action [35, 36]. For instance, they phosphorylate serine of IRS-1 and reduce insulin-induced tyrosine phosphorylation [35, 36]. In other respects, they are also responsible for the down-regulated protein related to glucose metabolism, such as above-mentioned GLUT4 [37] and PPARγ [38]. IL-6 also occupies a pivotal position in IR and T2DM. It increases serine phosphorylation and inhibits the expression of GLUT4 to impair glucose consumption [39]. FC suppressed these inflammatory cytokines, which might account for ameliorating glucose homeostasis and improving IR.

**Fig. 7:**
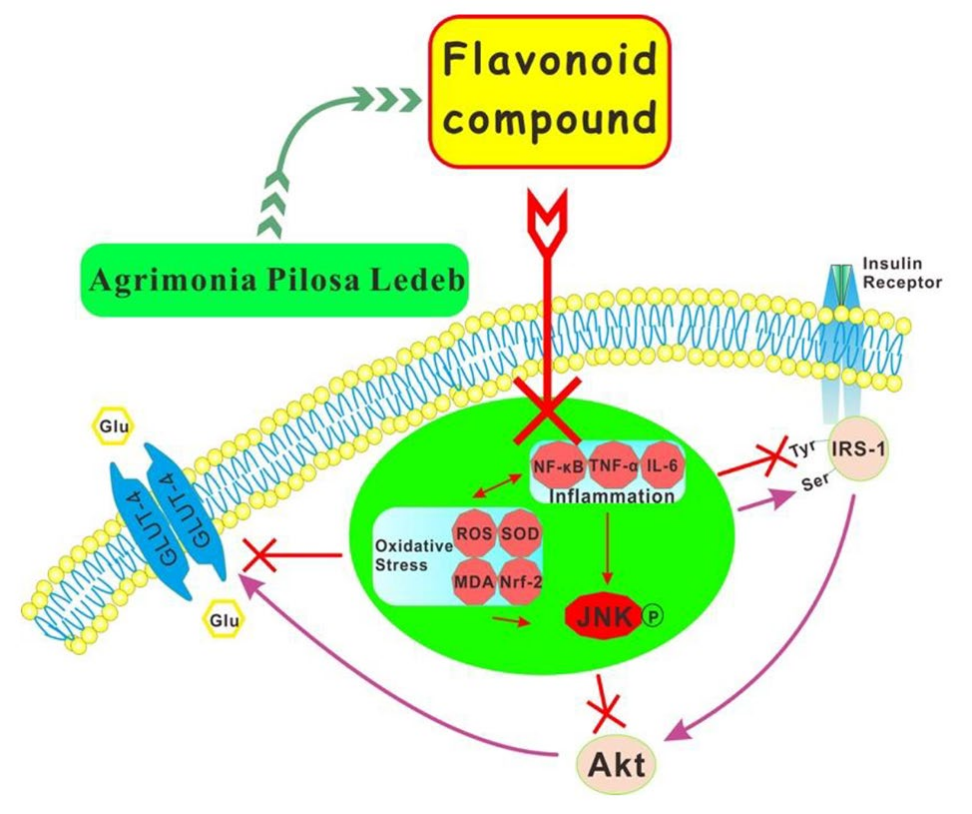
Effect of FC on JNK/PI3K/Akt pathway to prevent IR.

Is there a cross talk between reducing oxidative stress and suppressing inflammation by FC to improve IR? We attempted to answer this by analyzing mitogen-activated protein (MAP) kinases. JNK is activated in response to various extracellular stimuli and cellular stresses, such as ROS, TNF-α and FFAs which are known to be activated and accelerated in IR. Moreover, mounting evidence [25] indicates that activation of JNK is the key factor of mediating IR. It can bind to the serine of IRS-1 to phosphorylate IRS-1 and suppress the tyrosine phosphorylation which induce impaired insulin signal pathway. It has been demonstrated that the activation of JNK is essential for oxidative stress and inflammation, while inhibiting JNK can prevent IR [40, 41]. Therefore, oxidative stress, inflammation and JNK are closely related. Akt, a downstream target of PI3K, is triggered by the activation of insulin receptor in insulin signal pathway and facilitates the uptake of glucose via GLUT [7]. Besides, the increased PI3K and activated Akt also increase the insulin-stimulated glucose disposal [7]. Therefore, blocking JNK/PI3K/Akt pathway is an approach to stopping oxidative and inflammatory insult on the initiation of IR and T2DM (Fig. 7). In this study, FC alleviated this signal pathway forcefully to keep insulin sensitivity, which makes FC a promising alternative to prevent IR.

## Conclusions

Oxidative stress and inflammation are main factors in IR and T2DM. Based on our results, FC from *Agrimonia pilosa Ledeb* could ameliorate the glucose metabolism in IR model. Furthermore, it improved insulin sensitivity of adipocytes and adipose tissue. We found FC alleviated the disturbed oxidative homeostasis and also decreased the expression and secretion of inflammatory cytokines via JNK/PI3K/Akt pathway. To summarize, as an agent of anti-oxidative stress and anti-inflammation, FC from *Agrimonia pilosa Ledeb* has a good prospect to be a health care product or natural drug for preventing IR.

### Electronic supplementary material

Below is the link to the electronic supplementary material.

Supplementary Material 1

## Data Availability

All data generated or analyzed during this study are included in this published article.

## Declarations

### Statement

We have permission to collect *Agrimonia pilosa Ledeb* for basic research.

## Abbreviations

IR: insulin resistance
FC: flavonoid component
ROS: reactive oxidative species
SOD: superoxide dismutase
MDA: malondialdehyde
IRS-1: insulin receptor substrate-1
Nrf2: NF-E2-related factor 2
JNK: c-Jun N-terminal kinase
FBS: fetal bovine serum
BSA: bovine serum albumin
IBMX: 3-isobutylmethylxanthine
DEX: dexamethasone
2-NBDG: 2-(N-[7-Nitrobenz-2-oxa-1, 3-diazol-4-yl)Amino]-2-Deoxyglucose
BCS: bovine calf serum
LFD: low-fat diet
HFD: high-fat diet
WAT: white adipose tissue
