# Supplementary material for: Flavonoid compound from Agrimonia pilosa Ledeb improves adipose insulin resistance by alleviating oxidative stress and inflammation
Source: BMC Complement Med Ther. 2023 Sep 14;23:322. doi: 10.1186/s12906-023-04114-5 (PMC10503054; doi:10.1186/s12906-023-04114-5)
Supplement: Supplementary file 1 — Supplementary Material 1 [file 12906_2023_4114_MOESM1_ESM.docx]

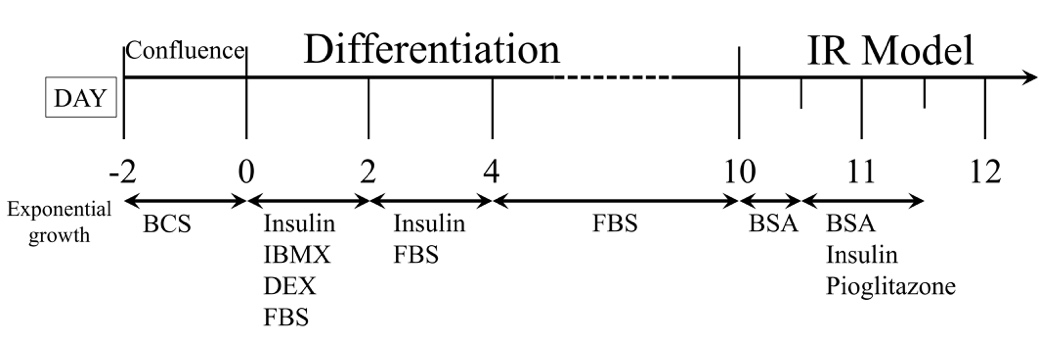


Figure S1. The protocol of 3T3-L1 differentiation and IR model. IR, insulin resistance; BCS, bovine calf serum; IBMX, 3-isobutylmethylxanthine; DEX, dexamethasone; FBS, fetal bovine serum; BSA, bovine serum albumin.


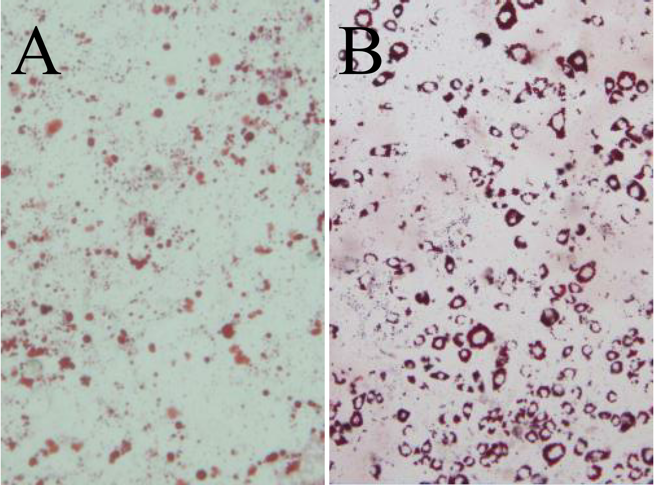


Figure S2. The picture of Red Oil of 3T3-L1 pre-adipocytes (a) and adipocytes (b). The degree of 3T3-L1 preadipocytes differentiation was assessed by Oil Red O staining of accumulated lipids.


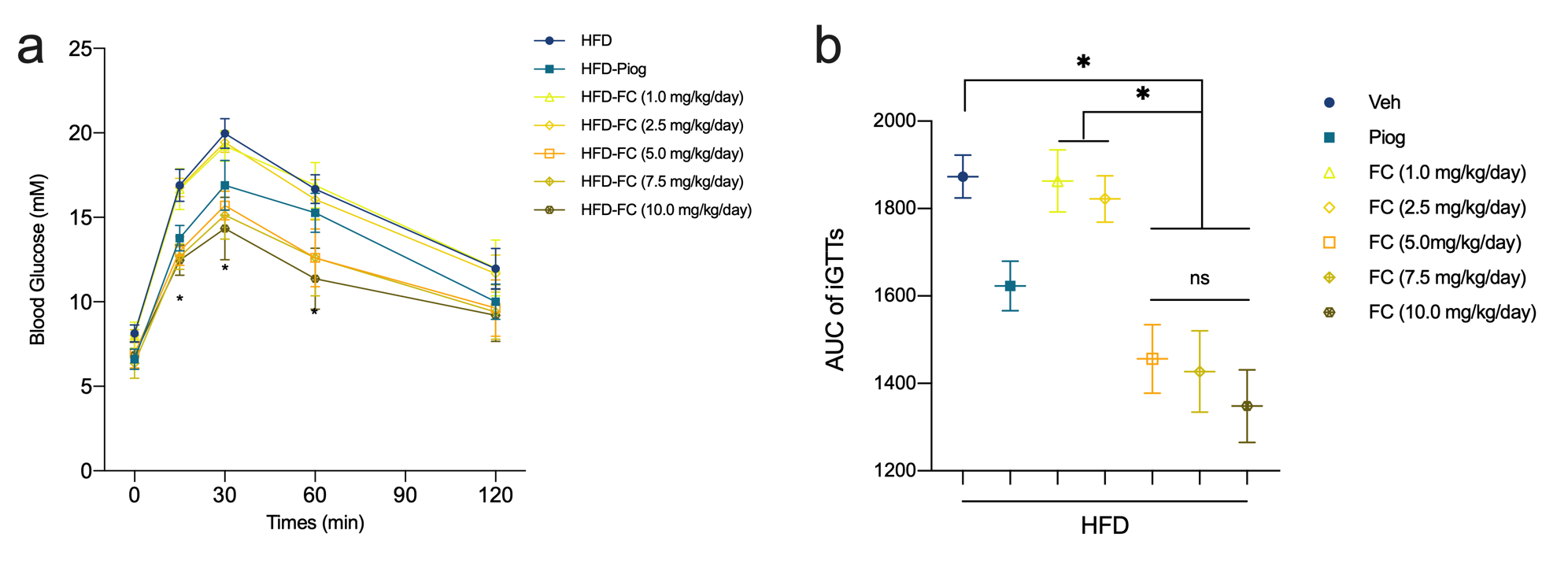


Figure S3. FC recovered the disturbed glucose metabolism. (A) iGTTs were performed after starvation overnight after 20 weeks. (B) AUC of iGTTs was calculated. Data are expressed as mean ± SEM (n=6). * *p*<0.05, ** *p* <0.01 and *** *p* <0.005. HFD, high fat diet; LFD, low fat diet; iGTTs, intra-peritoneal glucose tolerance tests; AUC, area under curve.


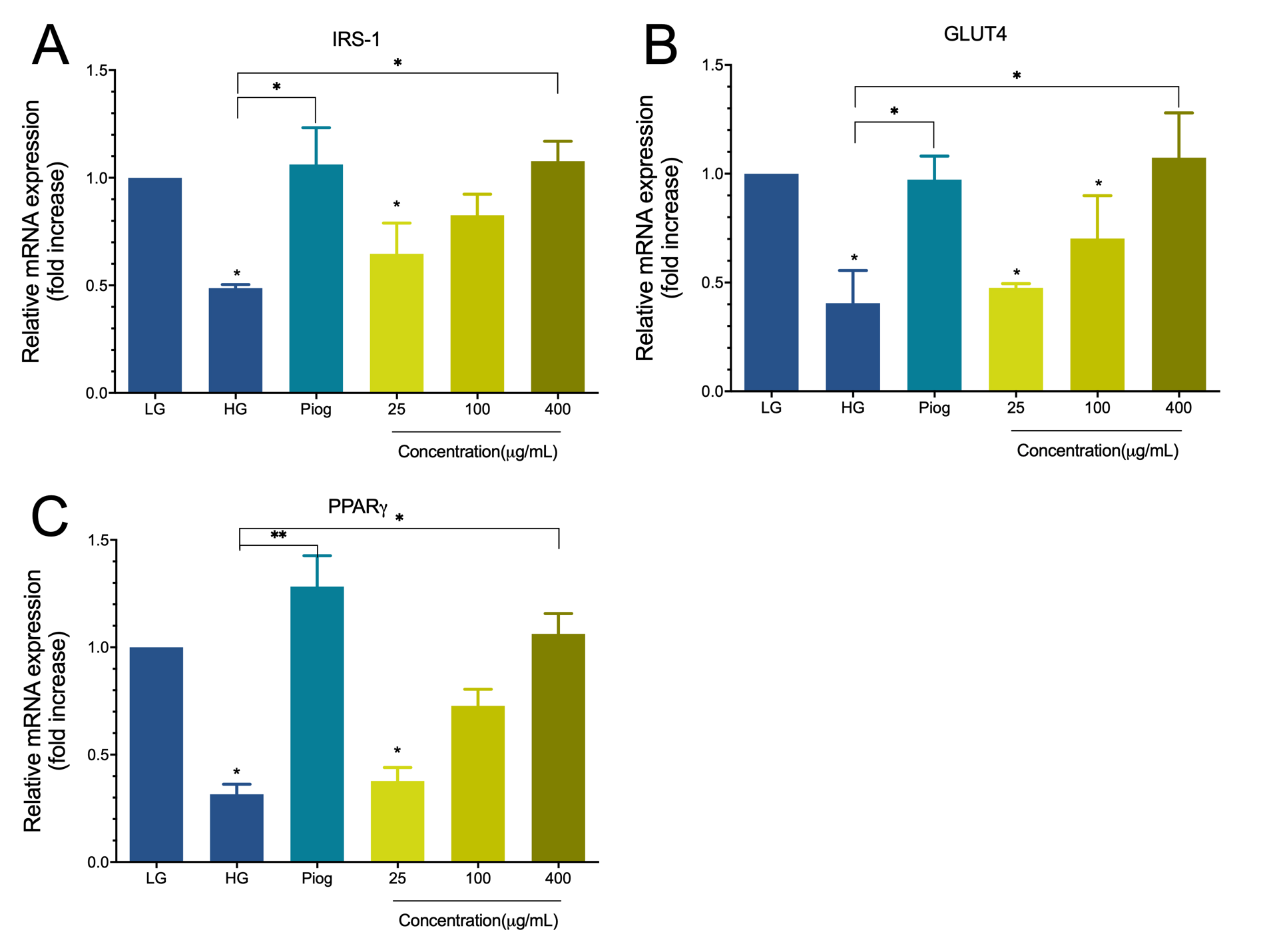


Figure S4. Effect of FC on the main relative genes related to insulin signal pathway of 3T3-L1 adipocytes with IR. Data are expressed as mean ± SEM (n=6). * *p* <0.05, ** *p* <0.01 and *** *p* <0.005.


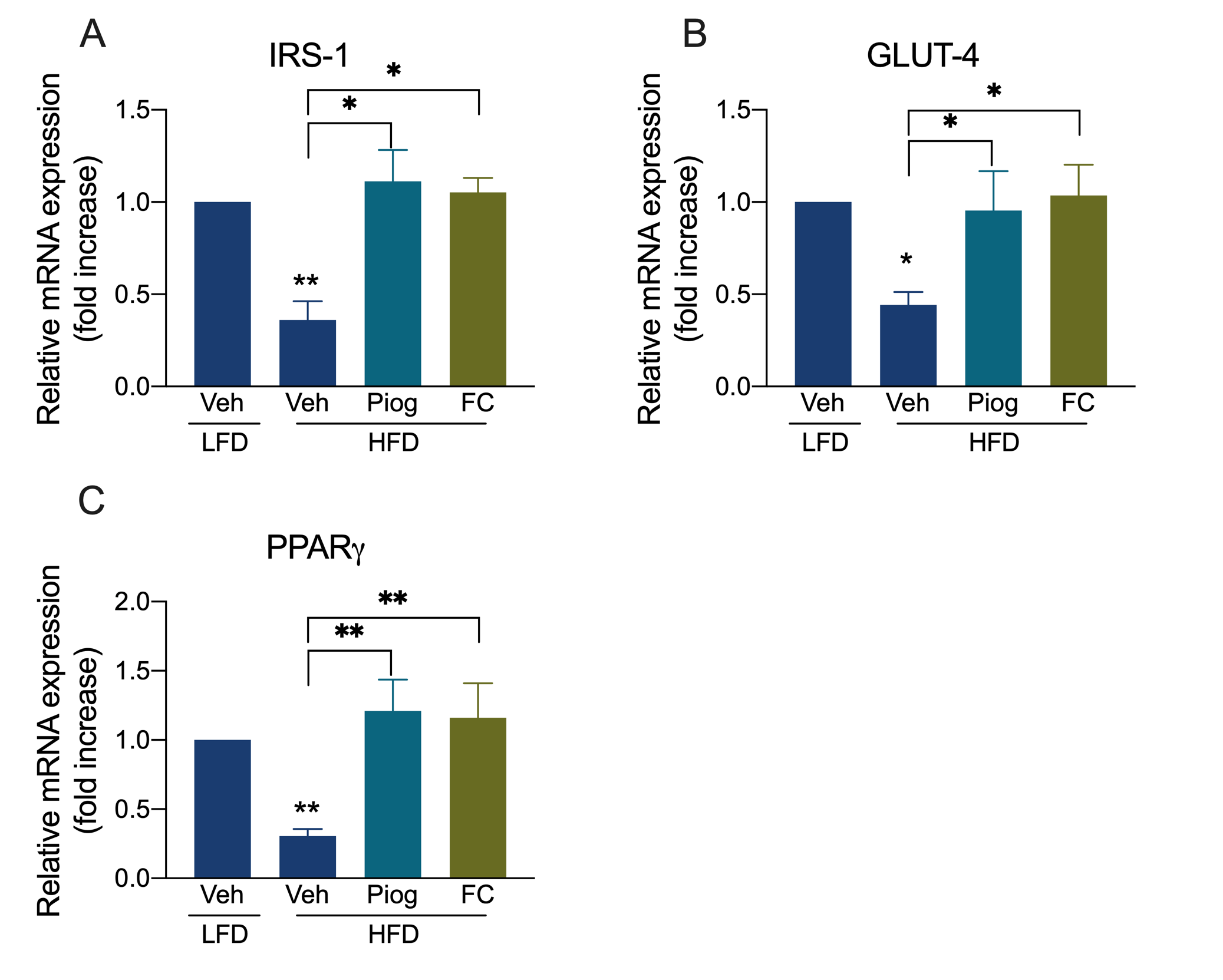


Figure S5. Effect of FC on the main relative genes expression of insulin signal pathway in C57BL/6 mice IR model. Data are expressed as mean ± SEM (n=6). * *p* <0.05, ** *p* <0.01 and *** *p* <0.005.


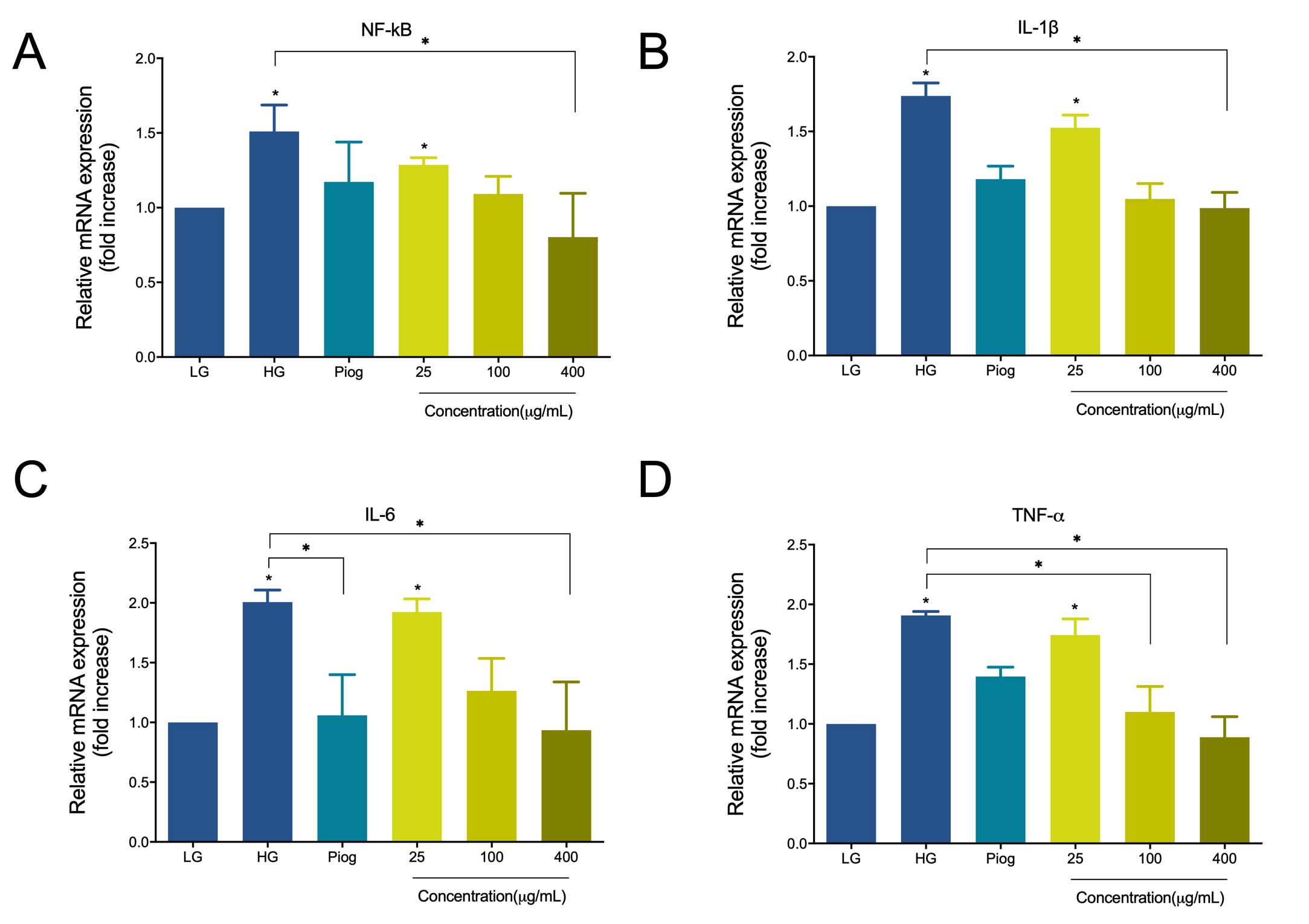


Figure S6. Effect of FC on genes expression of inflammatory cytokines in adipocytes. Data are expressed as mean ± SEM (n=6). * *p* <0.05, ** *p* <0.01 and *** *p* <0.005.


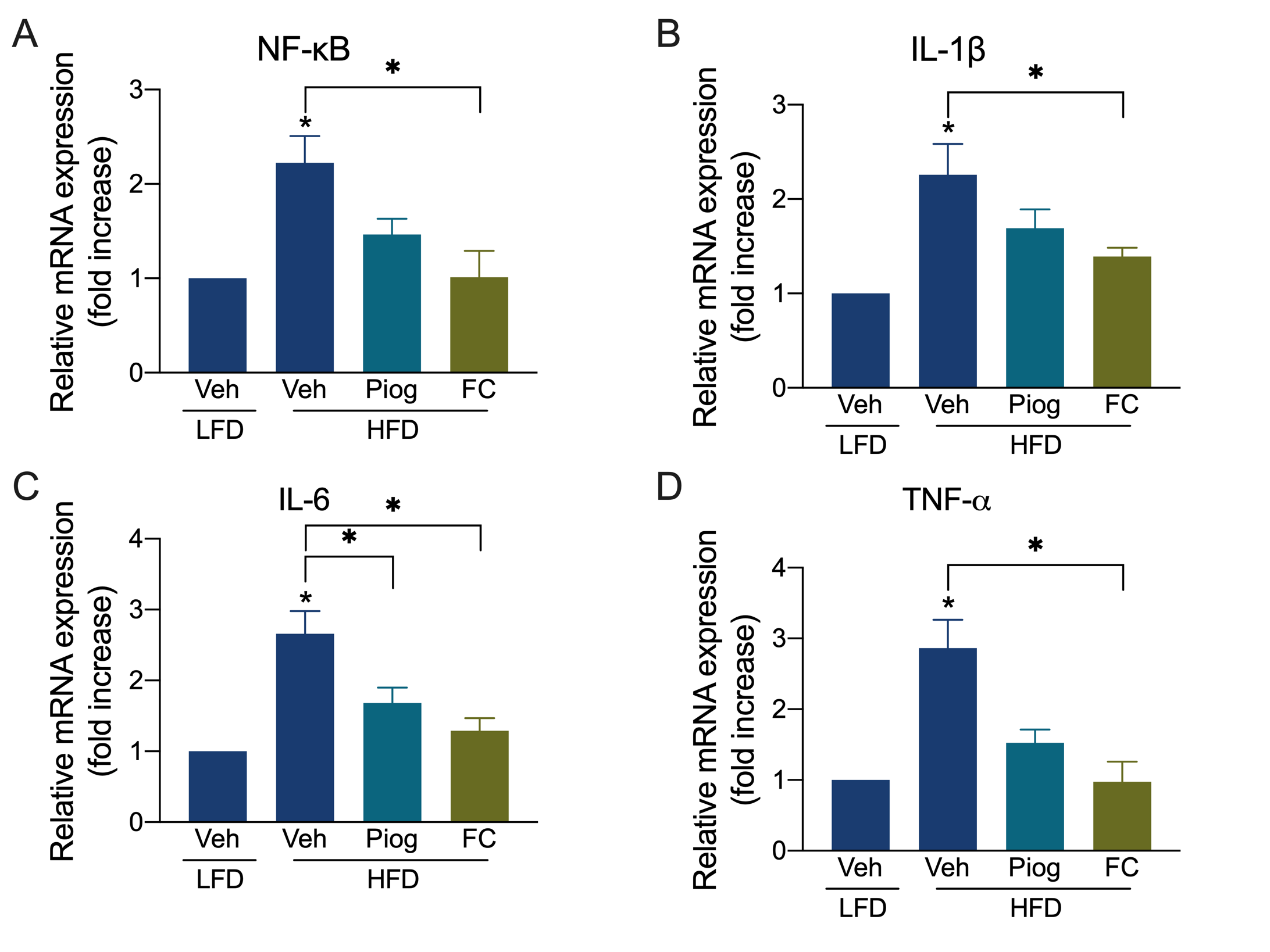


Figure S7. Effect of FC on genes expression of inflammatory cytokines in adipose tissue. Data are expressed as mean ± SEM (n=6). * *p* <0.05, ** *p* <0.01 and *** *p* <0.005.
